# Supplementary material for: Responsive core-shell DNA particles trigger lipid-membrane disruption and bacteria entrapment
Source: Nat Commun. 2021 Aug 6;12:4743. doi: 10.1038/s41467-021-24989-7 (PMC8346484; doi:10.1038/s41467-021-24989-7)
Supplement: Supplementary file 1 — Supplementary Information [file 41467_2021_24989_MOESM1_ESM.pdf]

Supplementary Information: Responsive core-shell DNA particles trigger lipid-membrane disruption and bacteria entrapment

Michal Walczak,[1] Ryan A. Brady,[2] Leonardo Mancini,[1] Claudia Contini,[3] Roger Rubio-Sánchez,[1] William T. Kaufhold,[1,3] Pietro Cicuta,[1] and Lorenzo Di Michele[3,1,*]

[1]*Biological and Soft Systems, Cavendish Laboratory, University of Cambridge, JJ Thomson Avenue, Cambridge CB3 0HE, United Kingdom*

[2]*Department of Chemistry, Faculty of Natural and Mathematical Sciences, King's College London, London SE1 1DB, United Kingdom*

[3]*Molecular Sciences Research Hub, Department of Chemistry, Imperial College London, London W12 0BZ, United Kingdom*

(Dated: May 29, 2021)

SUPPLEMENTARY DISCUSSION 1: DIFFUSION–REACTION GROWTH MODEL

The dependence of the hydrodynamic size of particles on the growth time t_{g} (Fig. 2**a**) was fit to a model for the diffusion-reaction-limited particle growth, as described in chapter 4 of Ref. [1]. While simpler diffusion-limited (Lifshitz–Slyozov–Wagner theory) or reaction-limited models have been successfully applied in some cases [2], in most situations, including ours, the mixed reaction-diffusion model is required to fit the data [3]. Briefly, the change in particle size r is generally described by

$$\frac{\mathrm{d}r}{\mathrm{d}t} = \frac{2\sigma V_m^2 c_\infty}{RT(1/D + 1/k_d r)} \frac{(1/r_b - 1/r)}{r}, \tag{1}$$

where σ is the interfacial energy of the particle, k_d is the first-order rate constant of the deposition reaction and r_b is the critical nucleus size. Furthermore, in Eq. 1, V_m, c_∞ and D are respectively the molar mass, bulk concentration and diffusion coefficients of the molecular constituents of the particles, in our case ssDNA building blocks and cholesterol-DNA micelles. R is the ideal gas constant.

It has been shown that for both the diffusion-limited case and the reaction limited case the r/r_b ratio is constant and equal to 1 (diffusion)[4], and 8/9 (reaction)[5]. Assuming that these conditions can be extended to the mixed diffusion-reaction model, and defining $r/r_b = K'$ [3], Eq. 1 can be expressed as

$$\frac{\mathrm{d}r}{\mathrm{d}t} = \frac{2\sigma V_m^2 c_\infty (K' - 1)}{RTr^2(1/D + 1/k_d r)}, \tag{2}$$

and subsequently integrated to derive:

$$t = Ar^3 + Br^2 + C, \tag{3}$$

where $A = RT/6D\sigma V_m^2 c_\infty (K' - 1)$, $B = RT/4k_d\sigma V_m^2 c_\infty (K' - 1)$ and C is a constant.

Equation 4 was used to fit the data for particles with both displaceable and non-displaceable corona using A, B and C as fitting parameters. Parameters A and B where allowed to vary freely while C was constrained to be negative due to the expected offset in particle size, caused by the presence of hydrophobic corona around the particle core. The best fit values, obtained by minimizing the square residuals divided by the variance of the experimental

measurement, are $A = 2.36 \times 10^{13}$ s m^{-3}, $B = 1.07 \times 10^{15}$ s m^{-2}, a $C = -12.7$ s for particles with displaceable corona and $A = 2.34 \times 10^{13}$ s m^{-3}, $B = 1.28 \times 10^{15}$ s m^{-2}, a $C = -11.06$ s for particles with non-displaceable corona.

Note that the model we use only describes growth by monomer addition and does not account for coalescence, which we expect to be a prominent process, particularly at large t_{g}. Additionally, in our experiments, the detected hydrodynamic size is also influenced by the presence of the corona decorating the particles at the end of the growth transient, whose thickness is expected not to depend on t_{g}. The resulting offset in particle size could be accounted for in the fits by the integration constant C.

SUPPLEMENTARY DISCUSSION 2: ESTIMATION OF PARTICLE POLYDISPERSITY FROM DDM DATA

The polydispersity of the core-shell particles was estimated from DDM data following Ref [6] for the samples prepared with growth time $t_g = 1800$ s. To this end, the fitting function for $\Delta I(q, \tau)$ in Eq. 1 of the main text was replaced with

$$\Delta I(q,\tau) = A(q)\left[1 - e^{-\tau/\tau_c(q)}\left(1 + \frac{\tau^2 \mu(q)}{2}\right)\right] + B(q). \tag{4}$$

Fitting was performed for each value of the wave-vector q by least-square minimization, optimizing over the wave-vector-dependent cluster relaxation time $\tau_c(q)$, and $\mu(q)$. $A(q)$ and $B(q)$ are functions dependent on the static scattering properties of the particles, the instrument optics and camera noise.

The polydispersity index (PDI) is given by $\mu\tau_c^2$, which corresponds to the first cumulant of the scattering distribution $G(\Gamma)$ [6]. Uncertainties in the values of $\tau_c(q)$ and $\mu(q)$ were estimated from the inverse Hessian matrix of the least squares fit, and propagated in quadrature to estimate the uncertainty in polydispersity.

Supplementary Figure 5**a** shows estimates of the polydispersity index as a function of q for particles obtained with growth time $t_g = 1800$ s, and monitored at room temperature at different time-points after assembly was completed. While PDI estimates fluctuate wildly outside the range of wave-vectors between (8×10^5 m^{-1}) and (1×10^6 m^{-1}), within this

range they are roughly consistent. An estimate of the polydispersity is extracted by the inverse-squared uncertainty weighted average of polydispersity index in this q-range.

In Fig. 5**b** we plot the mean polydispersity index as a function of time spent at room temperature after assembly. The PDI remains roughly unchanged with time, at around 0.5, indicating, along with the lack of substantial growth (Fig. 2b and 7), that the particles do remain stable after preparation. Polydispersity was estimated only for the relatively large particles produced with incubation time $t_g = 1800\,\mathrm{s}$, as $\Delta I(q,\tau)$ was excessively noisy for smaller particles to enable reliable polydispersity estimates.

Supplementary Table 1. **Oligonucleotide sequences.** Labels: CM – core motif, ICM – inner corona motif, OCM – outer corona motif, CS – cholesterol strand, NCS – non-cholesterol strand, Dα – domain α, Dα* – domain α*, Dβ – domain β, Dβ* – domain β*, IS - invading strand (trigger), FLUO - Fluorescein, TR - Texas Red, A647 - Alexa Fluor 647, T - toehold, TEG - Triethylene glycol.

Label	Oligonucleotide sequence (5' → 3')
CM.1	GGAGGTGGAGGTAGTGGATGGCTCGTCACTGCATACCGGCTGCTCAGAACATACATAACGCTTCGCCACGTCAAAC
CM.2	GGAGGTGGAGGTAGTGGATGGCGTTATGTATGTTCTGAGCTCGGACTCGCAAACGCCACGCTTTTTTTTTTTTTTT
CM.2 (FLUO)	GGAGGTGGAGGTAGTGGATGGCGTTATGTATGTTCTGAGC Fluorescein CGGACTCGCAAACGCCACGC
CM.2 (TR)	GGAGGTGGAGGTAGTGGATGGCGTTATGTATGTTCTGAGC Texas Red DT CGGACTCGCAAACGCCACGC
CM.3	GGAGGTGGAGGTAGTGGATGGCGTGGCGTTTGCGAGTCCGTCGTGTAAATGATGCGGTCGCTTCGCCACGTCAAAC
CM.4	GGAGGTGGAGGTAGTGGATGGCGACCGCATCATTTACACGTGCCGGTATGCAGTGACGAGCTTTTTTTTTTTTTTT
ICM.1	GCAATCGCCATCCGCGTCTGTTCGCATGATATCCGTGCTCCGGCTTCCAGCTCCG
ICM.2	CGGAGCACGGATATCATGCGTTCGCCGTAGAGCAGCAATTCGGCTTCCAGCTCCG
ICM.3	CGAATTGCTGCTCTACGGCGTTGGCCTCAGAGTCTGCGAACGGCTTCCAGCTCCG
ICM.4	CGTTCGCAGACTCTGAGGCCTTCGGTATGAGCCTGTGCAACGGCTTCCAGCTCCG
ICM.5	CGTTGCACAGGCTCATACCGTTCAATGGAGCCTTTACGGCACGCTTCCAGCTCCG
ICM.6	GTGCCGTAAAGGCTCCATTGTTCAGACGCGGATGGCGATTGCGTTTGACGTGGCG
ICM.6 (T)	GTGCCGTAAAGGCTCCATTGTTCAGACGCGGATGGCGATTGCGTTTGACGTGGCGGATTTG
OCM.1	GCAAGTGCTATGAGGTTGCGTTCCTGGTACTTTACATGTTCG
OCM.2	CGAACATGTAAAGTACCAGGTTGCATCGATGGTCGACGAACG
OCM.3	CGTTCGTCGACCATCGATGCTTGCCTTGACTGCCAATCAACG
OCM.3 (A647)	CGTTCGTCGACCATCGATGCTTGCCTTGACTGCCAATCAACG Alexa Fluor 647
OCM.4	CGTTGATTGGCAGTCAAGGCTTGCCGAAGTTGGAGCGGACCG
OCM.5	CGGTCCGCTCCAACTTCGGCTTGTGAGCATCTATCTCTAAAC
OCM.6	GTTTAGAGATAGATGCTCACTTCGCAACCTCATAGCACTTGCCGGAGCTGGAAGC
CS	CATCCACTACCTCCACCTCCAA TEG Cholesterol
NCS	CATCCACTACCTCCACCTCCAA
Dα	GTTTGACGTGGCG
Dα*	CGCCACGTCAAAC
Dβ	CGGAGCTGGAAGC
Dβ*	GCTTCCAGCTCCG
IS	CAAATCCGCCACGTCAAAC

Supplementary Table 2. **Sample composition.** Labels as in Supplementary Table 1. In rows where multiple oligos are indicated only one was used depending on the specific sample (*e.g.* cholesterolized/non-cholesterolized, fluorescent/non-fluorescent).

**Label**	**Concentration (μM)**
CM.1	1
CM.2/CM.2 (FLUO)/CM.2 (TR)	1
CM.3	1
CM.4	1
ICM.1	2
ICM.2	2
ICM.3	2
ICM.4	2
ICM.5	2
ICM.6/ICM.6 (T)	2
OCM.1	10
OCM.2	10
OCM.3/OCM.3 (A647)	10
OCM.4	10
OCM.5	10
OCM.6	10
CS/NCS	4
Dα	2
Dα*	2
Dβ	10
Dβ*	10
IS	20 (unless specified otherwise)

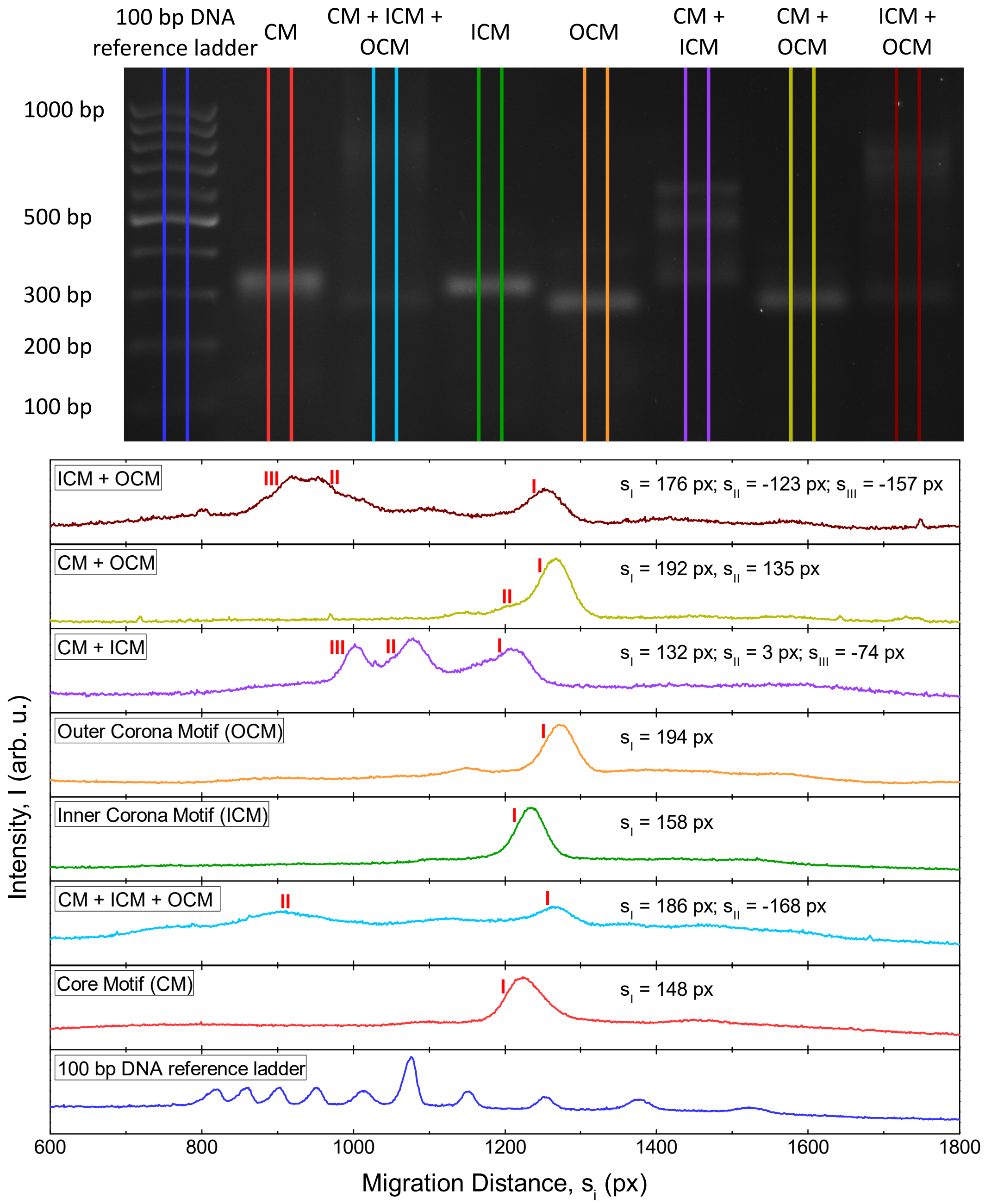

Supplementary Figure 1. **Agarose gel electrophoresis of non-cholesterolized nanostructures.** All individual motifs display a single bright band indicating the successful formation of DNA nanostructures. The one/two faint secondary bands suggest that a small percentage of the constructs aggregated or there was an excess of ssDNA left in solution. The CM + OCM sample shows two bands at the locations expected for the individual motifs indicating the lack of interaction (note that OCM is present in 10× excess compared to CM). As expected, all other samples show multiple bands or a smear band indicating the motif binding and the formation of larger constructs. For each of the sharp bands the relative migration distance (s_i, where i is a Roman numeral assigned to a band for the labelling purpose) was calculated by subtracting the migration distance of a selected sample band from the migration distance of a ladder band corresponding to a 500 bp structure.

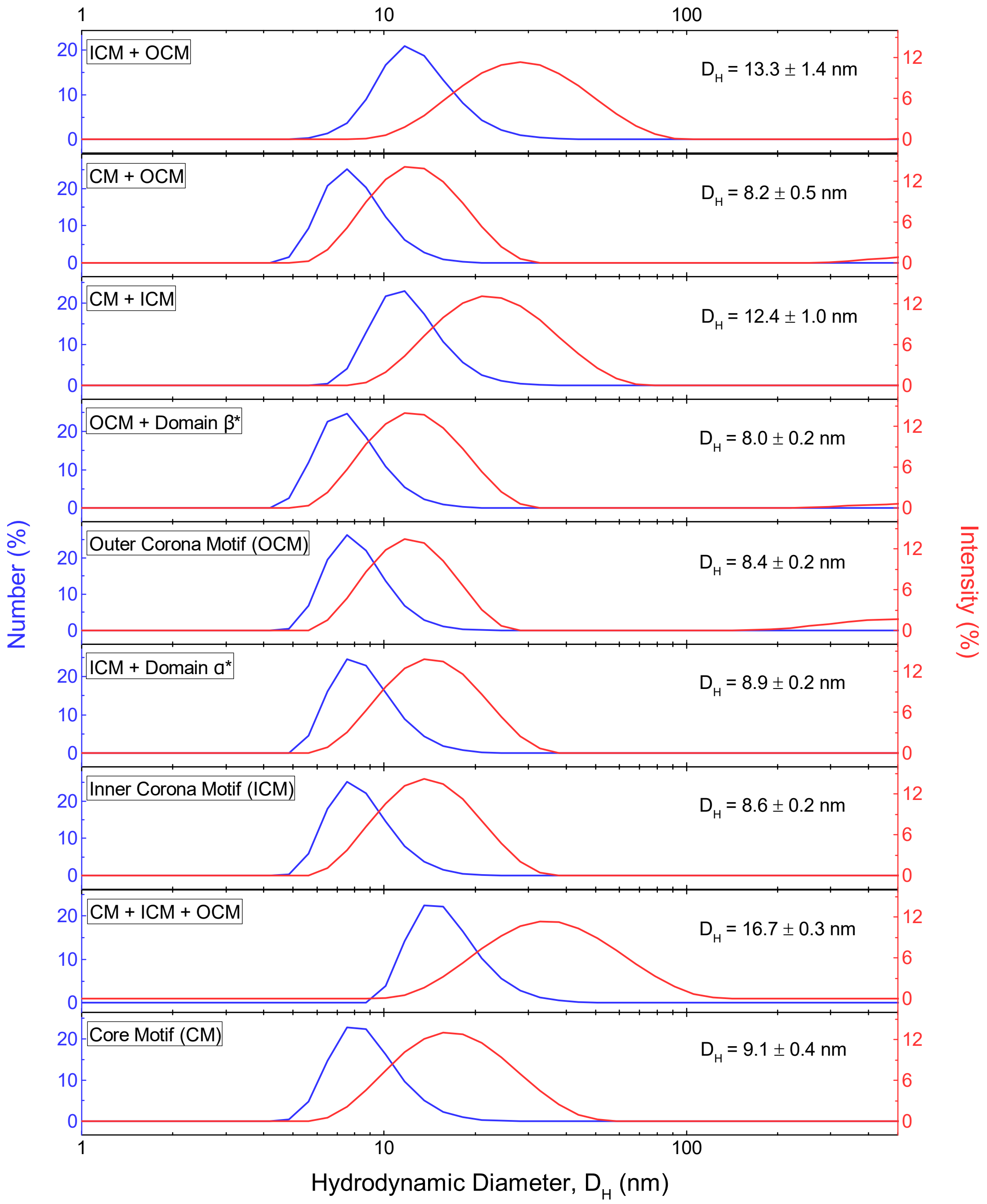

Supplementary Figure 2. **Dynamic light scattering of non-functionalized DNA motifs.** A shift to larger hydrodynamic diameters is observed in samples featuring motifs that are expected to connect in larger complexes (compare with AGE in Fig. 1). The hydrodynamic diameter was calculated from the averaged number signal of three data runs of twelve measurements each.

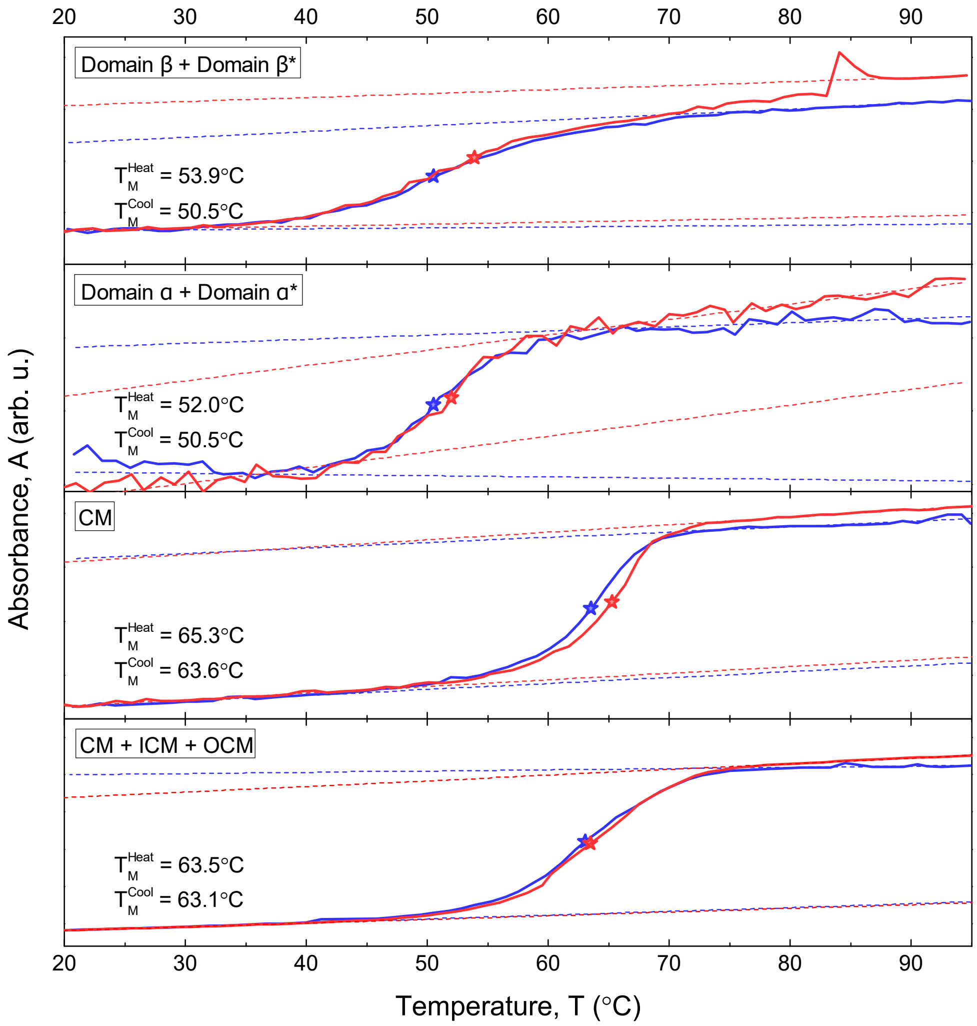

Supplementary Figure 3. **Experimental melting/hybridization curves of DNA motifs and overhang domains from UV absorbance.** Red and blue curves indicate data collected on heating and cooling, respectively. Melting temperatures ($T_{\mathrm{M}}^{\mathrm{Cool/Heat}}$, shown as stars) were determined as the intersection of the experimental data with the median of the two straight lines fitting the low- and high-temperature plateaus (shown as dashed lines)[7]. CM = core motif, ICM = inner corona motif, OCM = outer corona motif. Note how the the nanostructures (CM, CM+ICM+OCM) assemble/melt at higher temperature compared to the overhang domains α/α^* and β/β^*, which connect respectively CM to ICM and ICM to OCM. This separation in the melting temperatures is required for the two-step assembly procedure we rely on to construct the core-shell particles (see Fig. 1).

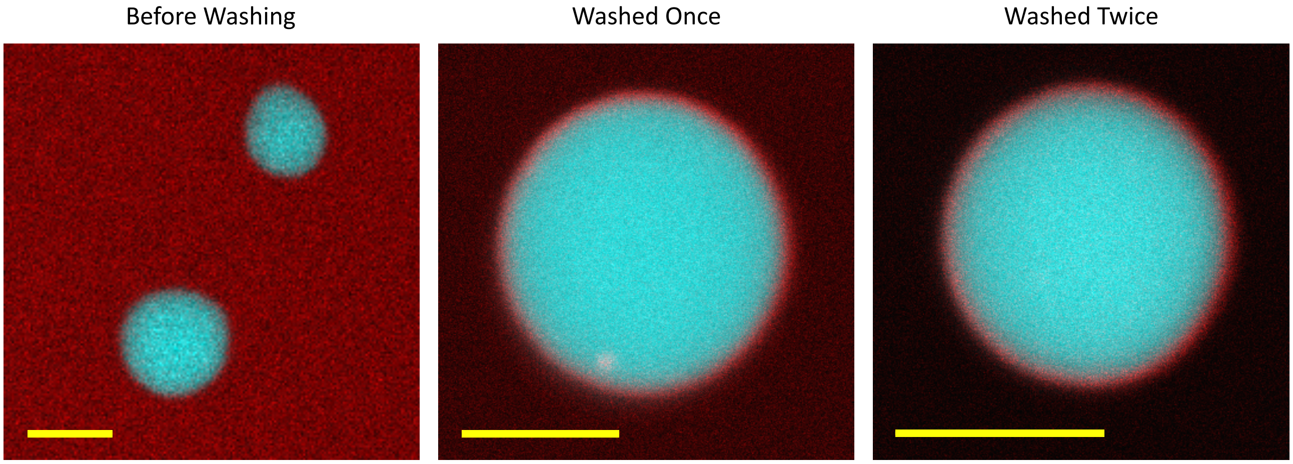

Supplementary Figure 4. **Confocal micrographs of large core-shell particles before and after removal of excess corona motifs.** After particle formation, a large number of dendrimer-like DNA aggregates formed by freely diffusing outer and inner-corona motifs remain in solution (Fig. 1). This is expected despite corona and core motifs are present in stoichiometric concentrations (Supplementary Table 1), since binding sites on the core motifs far from the aggregates' surface are expected to be inaccessible to corona motifs. Excess corona motifs can be successfully removed by supernatant replacement. Selected micrographs represent two independent experiments. Core motifs are labelled with fluorescein (cyan) and outer corona motifs with Alexa Fluor 647 (red). Scale bars are 5 μm.

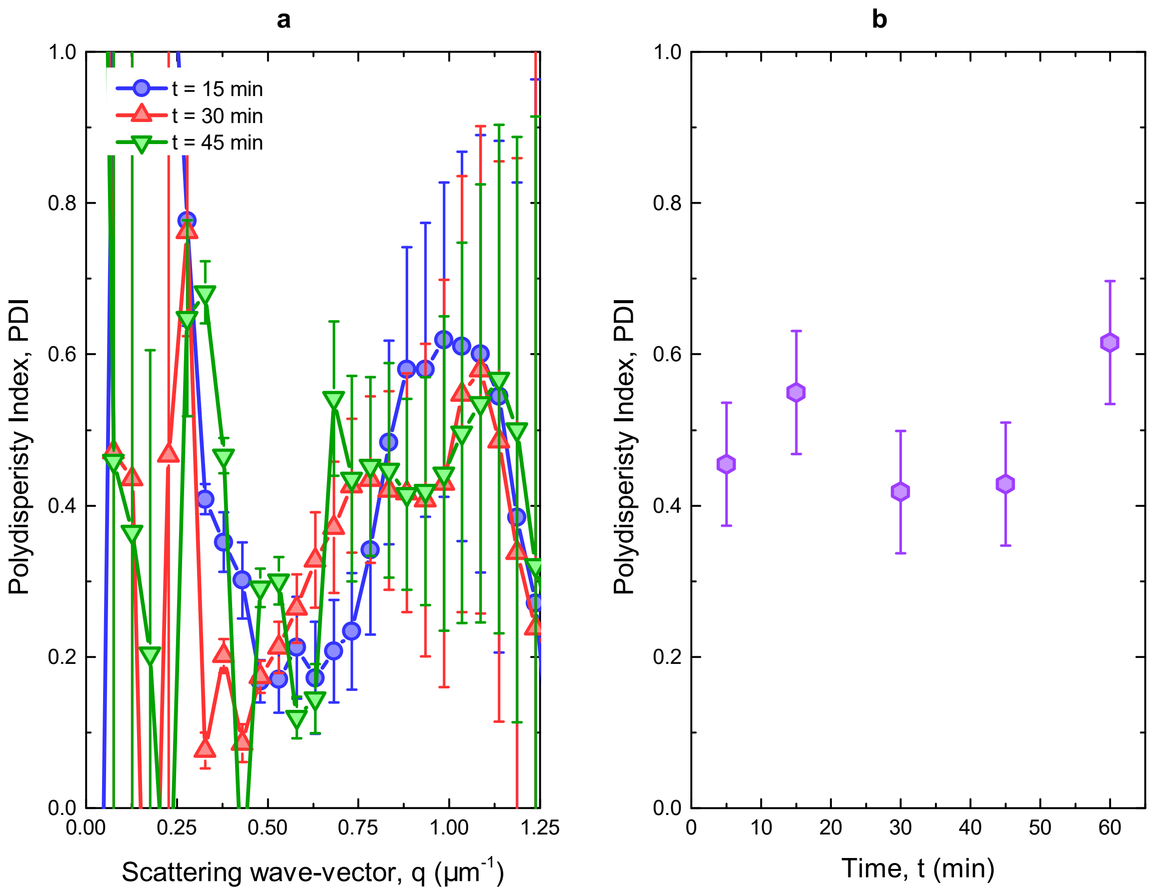

Supplementary Figure 5. **Particle size polydispersity index (PDI) as calculated from cumulant analysis of the DDM data. a.** PDI values are plotted as a function of the wave vector q for a sample incubated with growth time $t_g = 1800\,\mathrm{s}$ at various time points after preparation. **b.** q-averaged PDI as a function of the wait time after assembly. For a detailed explanation of the data processing and error estimation procedures see Supplementary Discussion 2. Data in panels **a, b** are a representation of three independent experiments.

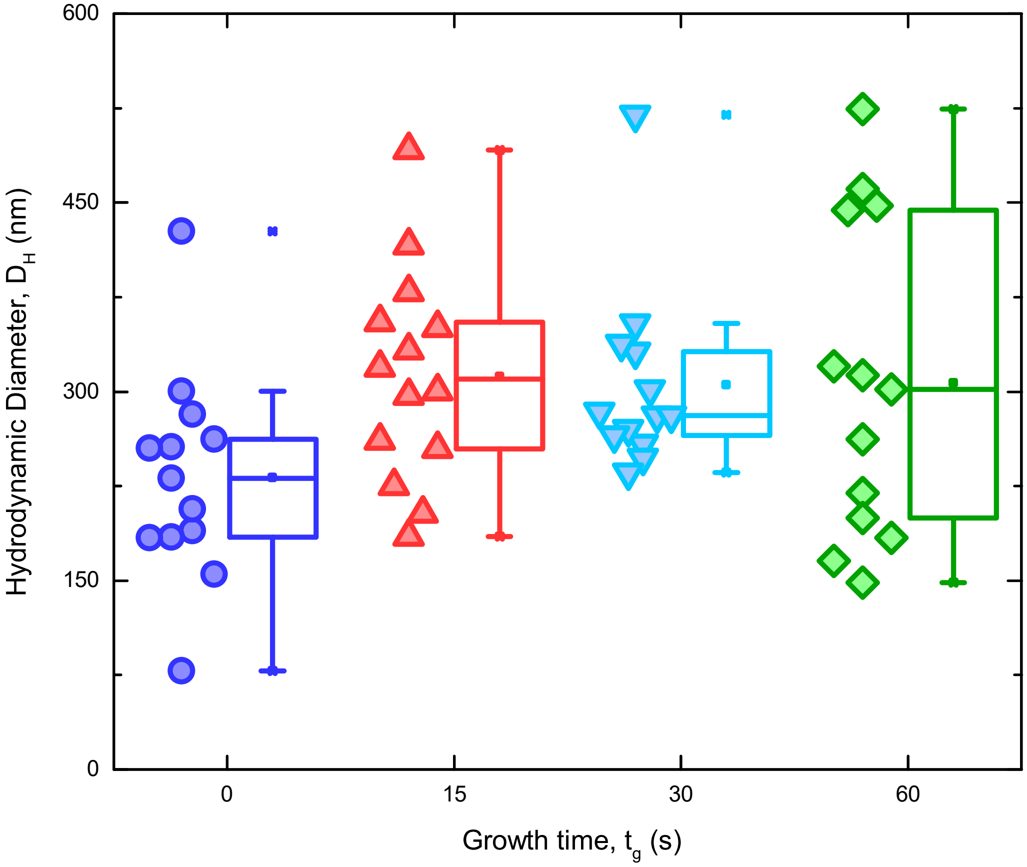

Supplementary Figure 6. **Hydrodynamic radius of protected particles prepared with various growth times as determined with DLS.** Particle size increases monotonically with growth time. Each experimental point represents the average value of D_H obtained from twelve data runs taken at room temperature. In box plots, the box is determined by the 75th percentile (upper bound), median (horizontal line) and 25th percentile (lower bound). The whiskers are determined by the 95th (top) and 5th (bottom) percentiles. The maxima, mean and minima are marked with squares (top to bottom).

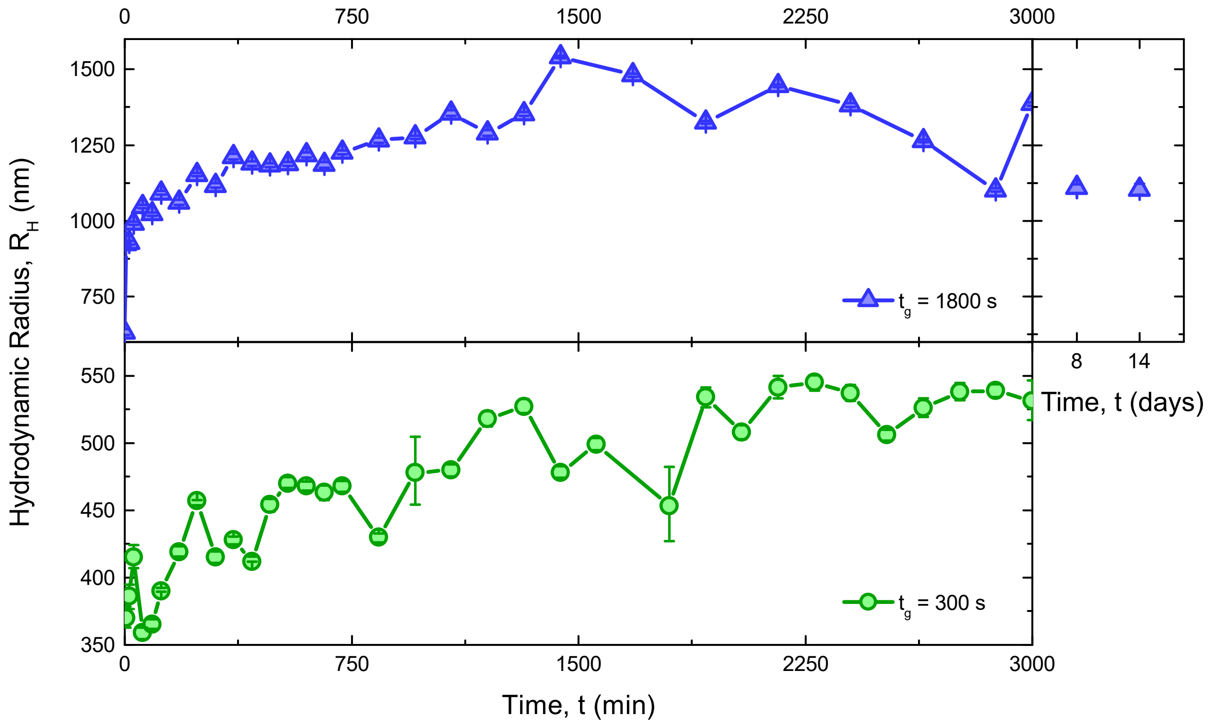

Supplementary Figure 7. **Long-term stability of protected particles as determined with DDM.** After a slight growth, hydrodynamic radius of particles as determined with DDM stabilizes, proving their stability against coalescence. R_H was measured for 50 h (3000 minutes) at room temperature. Two additional measurements were taken after 8 and 14 days for the sample assembled with t_g =1800 s. Each data point represents the value of R_H calculated from a single bright-field video and is plotted as R_H ± error value estimated from accuracy of the fit performed to extract the diffusion coefficient (see Methods). Compare with the curves collected for all tested growth times at shorter wait times in Fig. 2**b**.

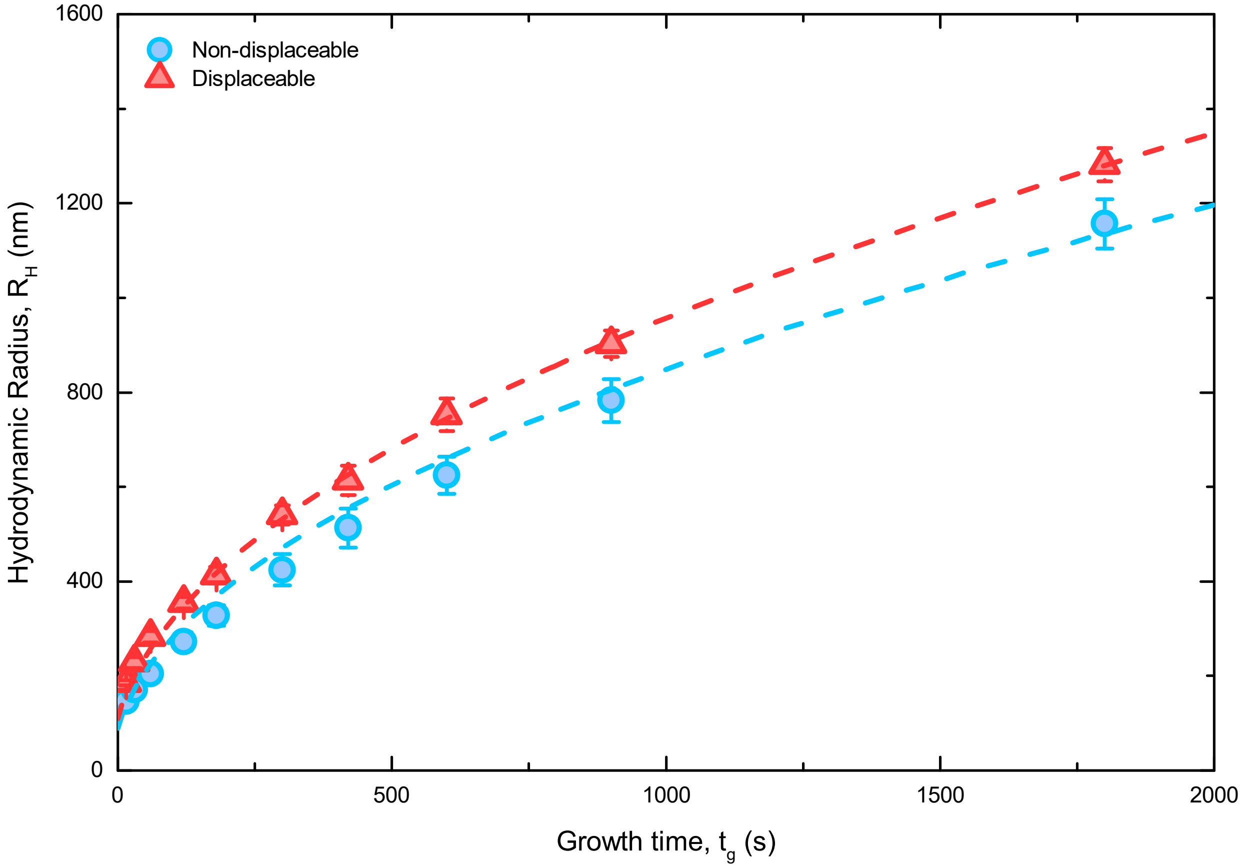

Supplementary Figure 8. **Hydrodynamic radius of protected particles with displaceable and non-displaceable corona.** Data analogous to those shown in Fig. 2**a**, but in which the hydrodynamic radius is averaged over the time interval covered in Fig. 2**b**, rather than just considering the end-point of the experiment (60 minutes). Note the very similar trends compared to those in Fig. 2**a**. Dashed lines are best fits to a diffusion-reaction growth model as summarised in Supplementary Discussion 1. Parameters: $A = 2.57 \times 10^{13}$ s m^{-3}, $B = 1.11 \times 10^{15}$ s m^{-2}, $C = -13.5$ s, and $A = 2.42 \times 10^{13}$ s m^{-3}, $B = 1.40 \times 10^{15}$ s m^{-2}, $C = -11.35$ s, for non-displaceable and displaceable, respectively.

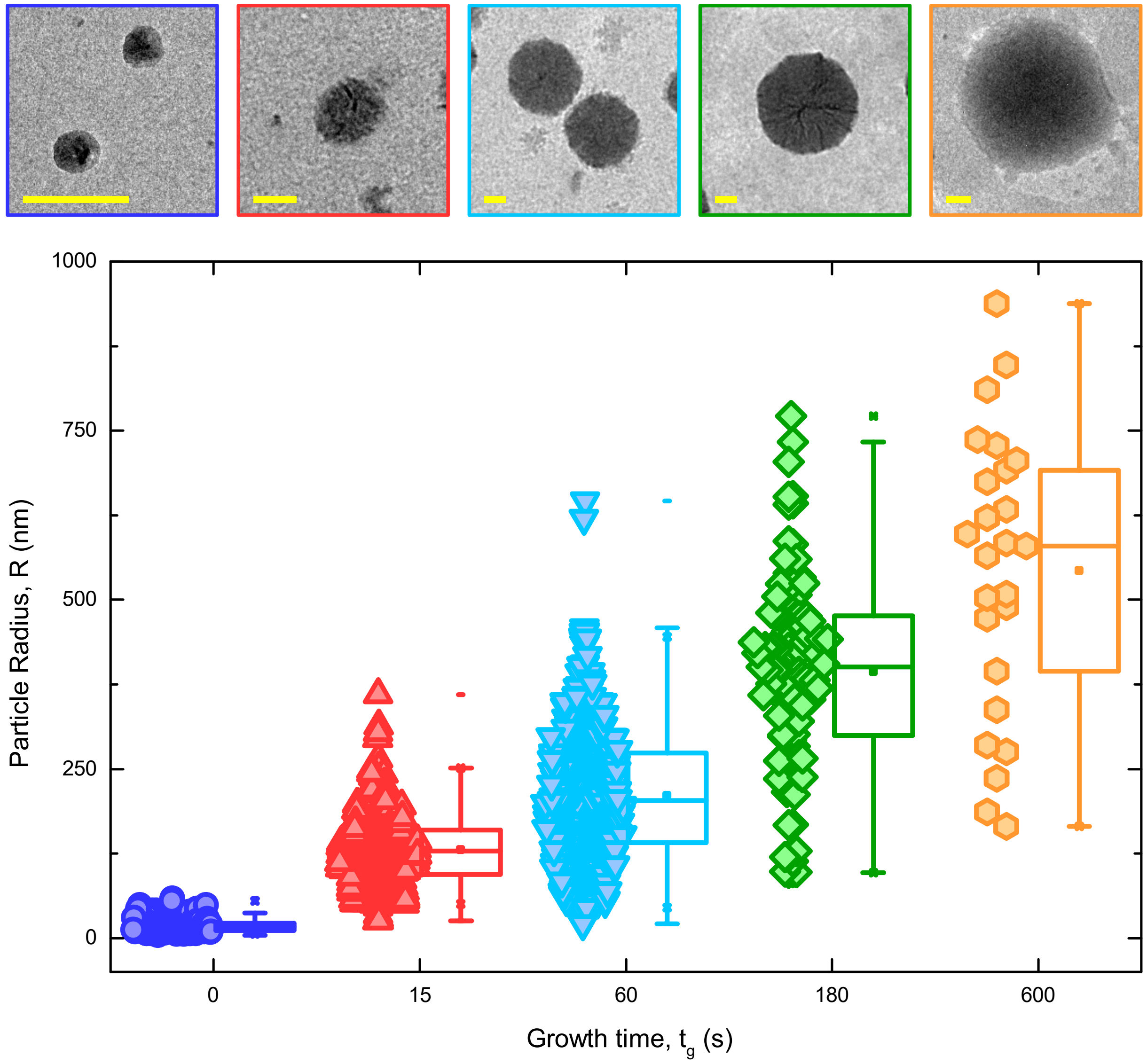

Supplementary Figure 9. **Size distribution of protected particles as a function of growth time as determined with TEM.** For all the studied growth times, the particle-size dependency on t_g follows a monotonic increase, which is consistent with the trend determined by DDM (Fig. 2**b**) and DLS (Fig. 6). The measured radii are in agreement with the values obtained by DDM. Each data-point represents a single particle whose size was determined from TEM micrographs acquired in a single experiment by taking four intensity profiles along the center of the particle, calculating a derivative of the obtained signal and measuring a distance between two local maxima corresponding to a border of the particle. The box in the box plots is determined by the 75th (upper bound) and 25th (lower bound) percentiles. The median is marked with a horizontal line. The whiskers are determined by the 95th (top) and 5th (bottom) percentiles. The maxima, mean and minima are represented with squares (top to bottom). Top: selected TEM micrographs from each sample. Scale bars are 200 nm.

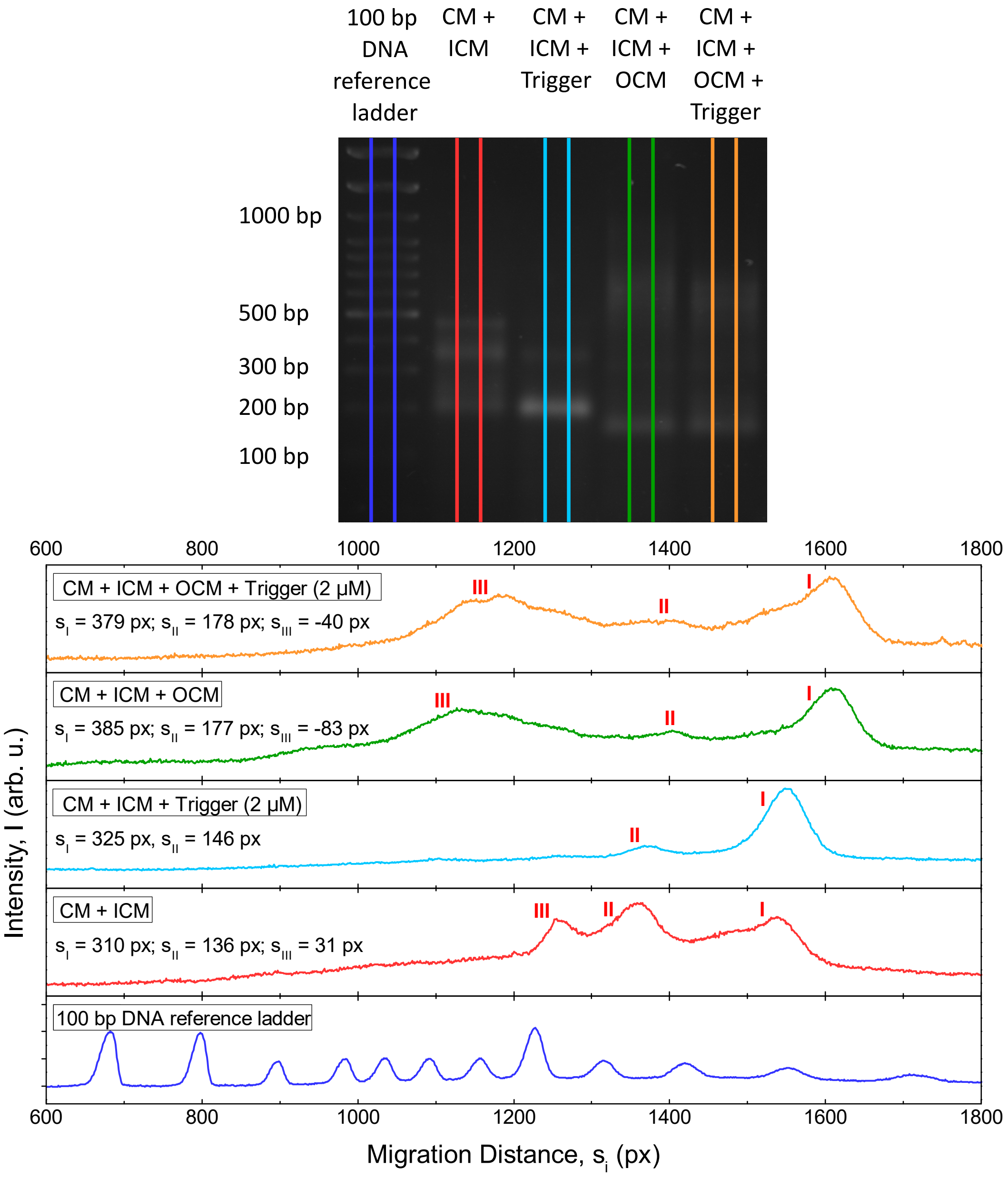

Supplementary Figure 10. **Trigger response of non-cholesterolized DNA nanostructures tested with AGE.** As expected, samples of core motifs (CM) and inner corona motifs (ICM) show two extra bands besides the one of the individual contstructs (I), the first is ascribed to complexes of one CM and one ICM (II) and the second to those of one CM and two ICM (III). These bands disappear almost completely after addition of the trigger strand, indicating the sought disassembly. Samples of CM, ICM and outer corona motifs (OCM) form a broad smear, mainly ascribed to large dendrimer-like aggregates of ICM and multiple OCM. These are not disrupted by the trigger and remain after its addition suggesting that AGE might not have the necessary resolution to distinguish the presence/absence of CM from these larger clusters. Trigger induced detachement of ICM-OCM dendrimers from CMs was confirmed with DLS in Fig. 11. For each of the sharp bands the relative migration distance was calculated (as in Fig. 1).

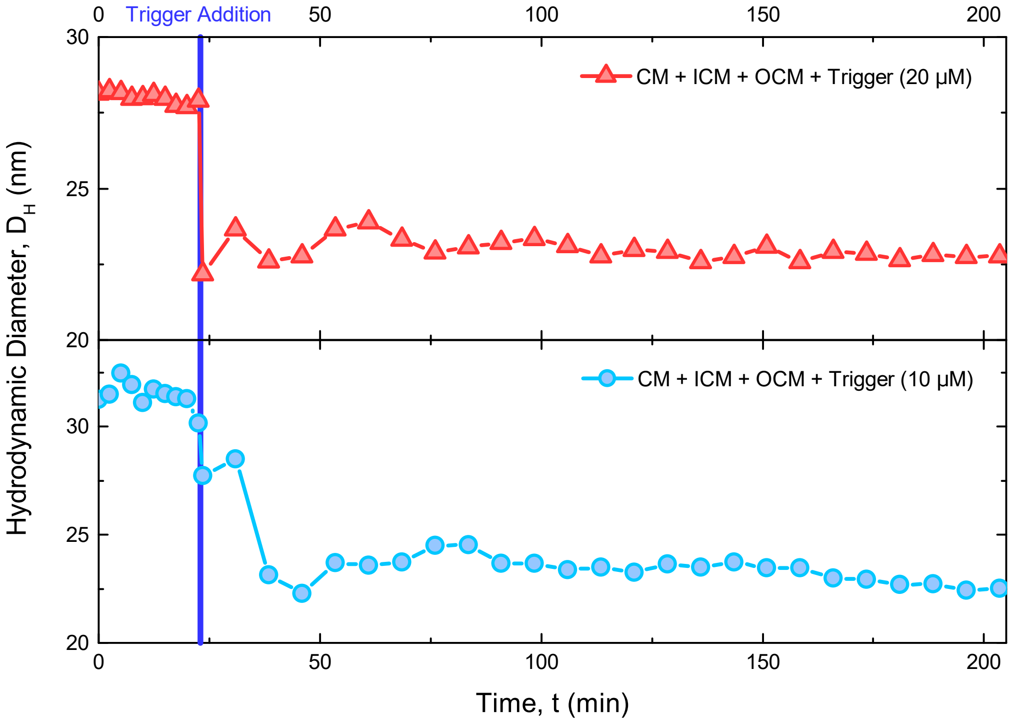

Supplementary Figure 11. **Trigger response of non-functionalized nanostructures tested with DLS.** Structures formed by core (CM), inner corona (ICM) and outer corona (OCM) motifs disassemble immediately after addition of trigger strand 10× in excess compared to the inner corona motifs. When the amount of added trigger is halved, the process completes in ∼15 min. Trigger addition is marked by a blue line.

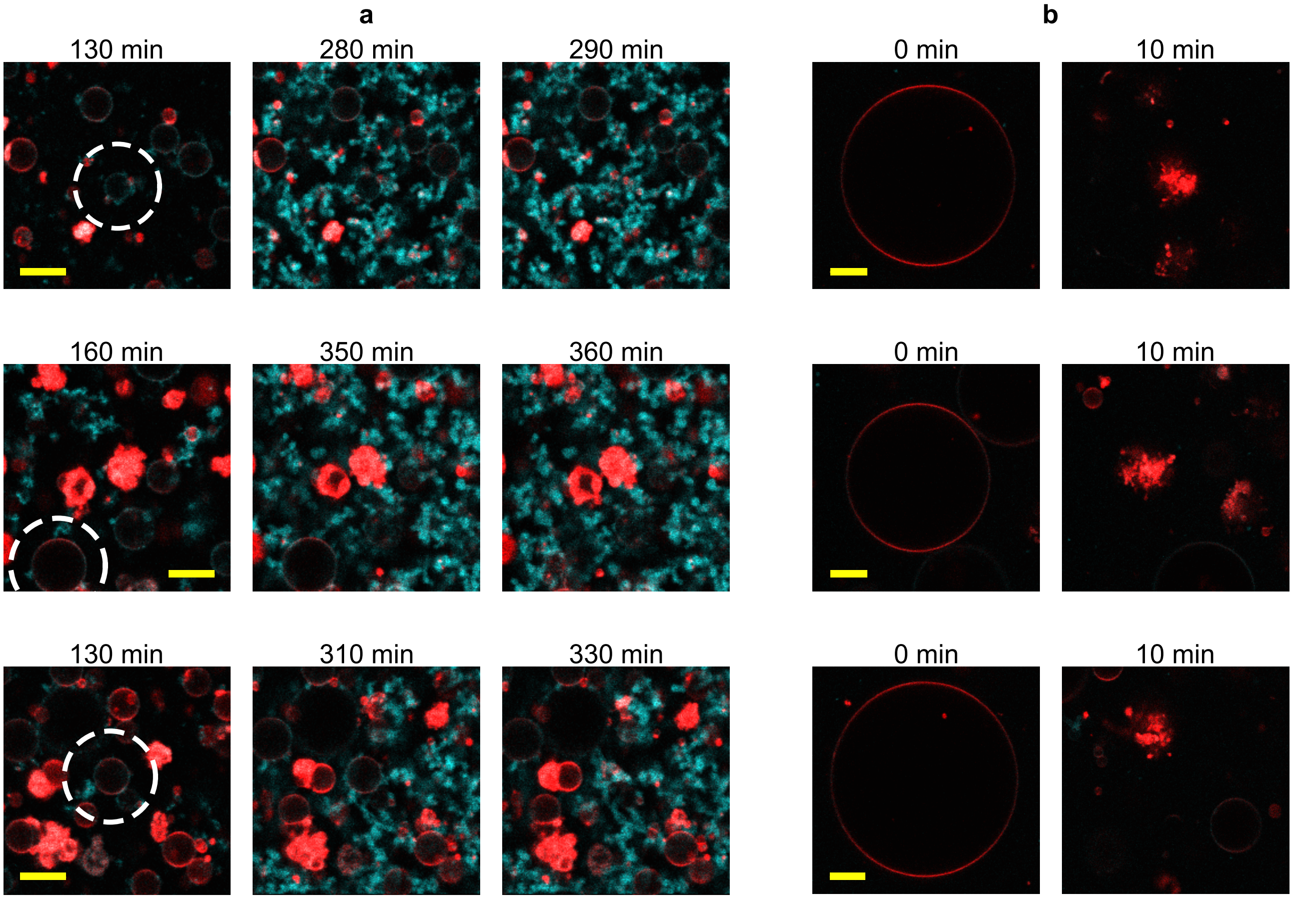

Supplementary Figure 12. **Confocal micrographs of vesicle rupture induced by triggered particles. a.** Three events where GUVs collapse after massive build up of DNA aggregates in their surrounding, resulting in them becoming entangled in a gel-like DNA network. The trigger strand, causing corona displacement from the particles, was added at time 0. **b.** Three examples where, instead, GUVs collapse earlier after the addition of the trigger strand without showing significant deposition of DNA on their surface. For both panels, image acquisition was preformed three times independently. Core motifs are labelled with fluorescein (cyan), and vesicles with Texas Red (red). Scale bars are 10 μm.

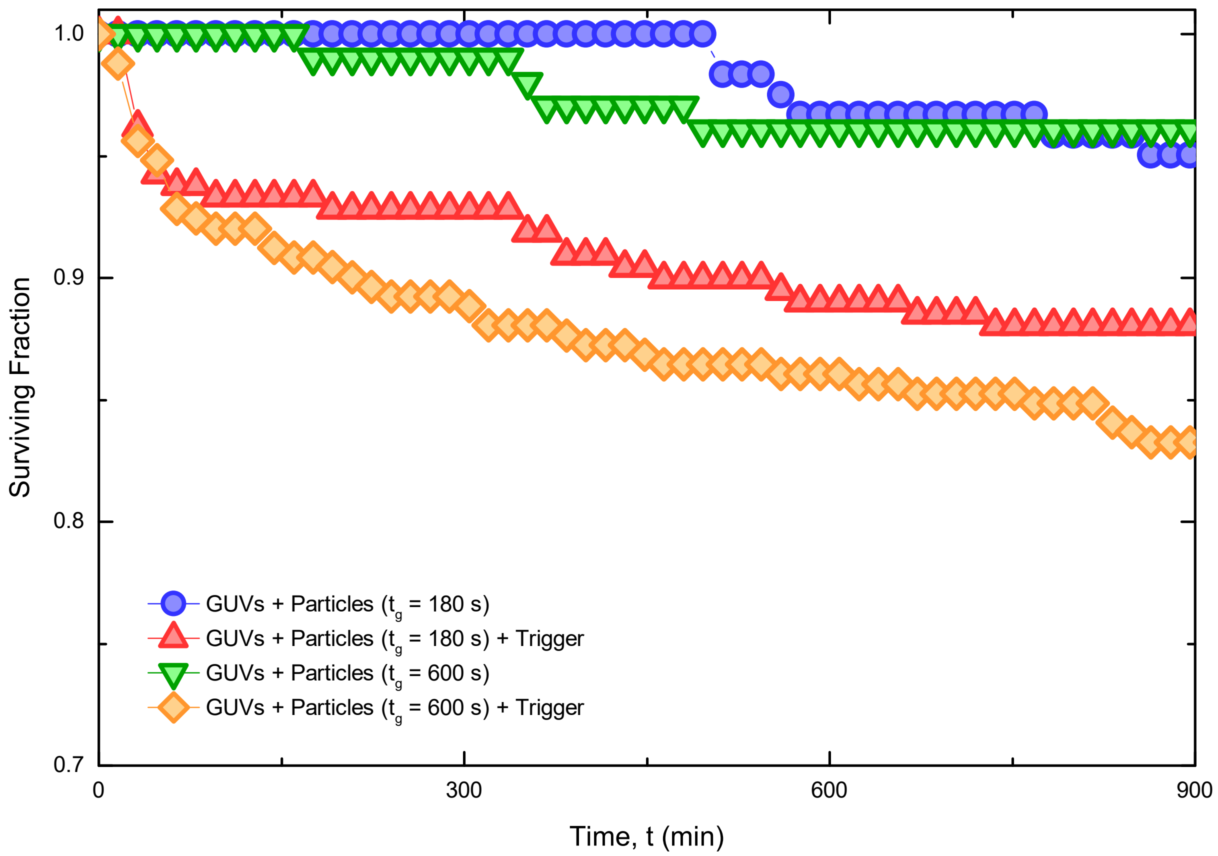

Supplementary Figure 13. **Giant Unilamellar Vesicle (GUV) rupture induced by particles of larger sizes.** Particles incubated for growth times (t_g) longer than 15 s have a significantly less disruptive influence on the membrane stability compared to their smaller counterparts (see Fig. 4**c**). This can be ascribed to the reduced diffusivity of larger particles, which leads to a limited DNA aggregation on GUVs surface at the initial stage of the experiment when membrane rupture is most prominent. The fraction of "surviving" GUVs was calculated from confocal micrographs obtained in a single experiment by taking the number of vesicles present in the field of view at $t = 0$ as reference. The (mass) concentration of GUVs was 3.12 ± 0.16 g L^{-1}.

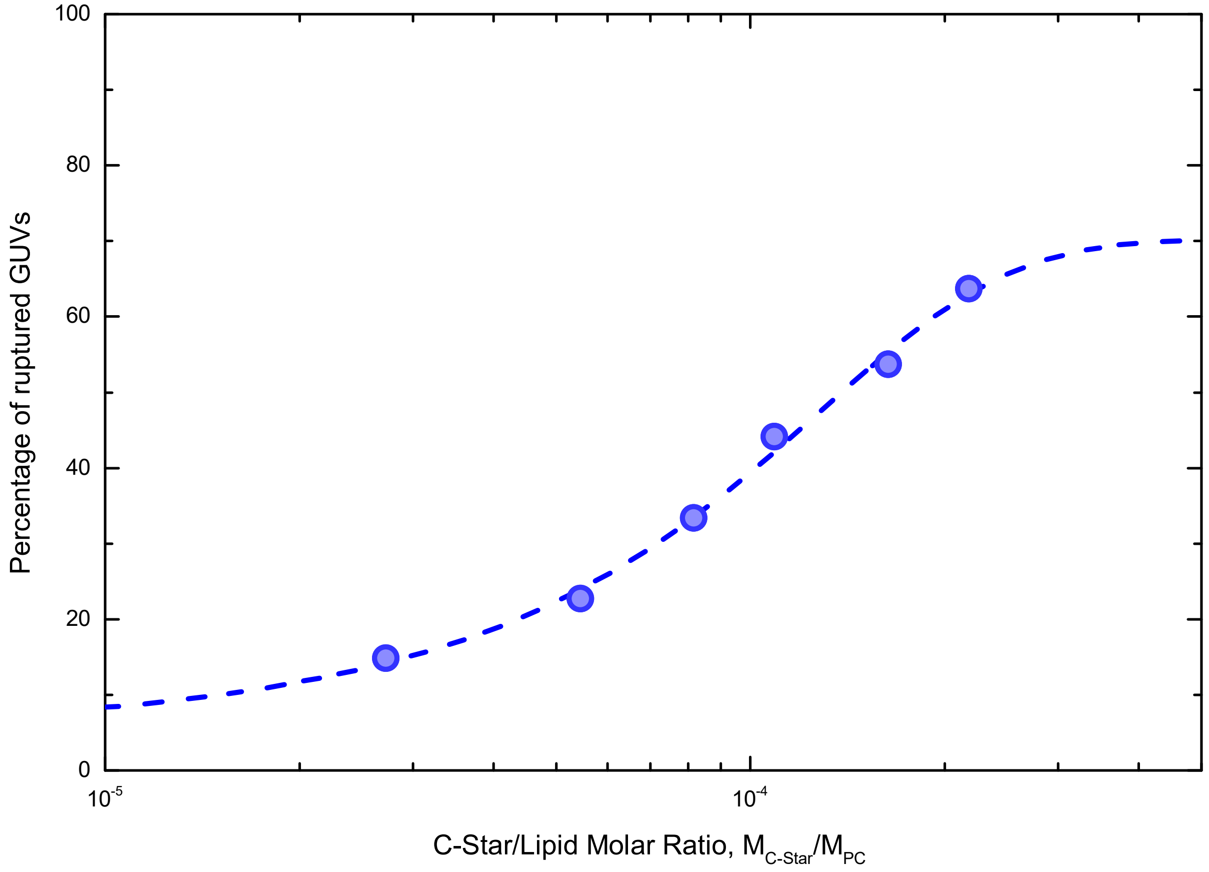

Supplementary Figure 14. **Dependence of the asymptotic fraction of disrupted Giant Unilamellar Vesicles (GUVs) on the C-Star/lipid molar ratio.** The percentage of ruptured GUVs was extracted from the data in Fig. 4**c** after 900 min from the trigger addition, and plotted as a function of C-Star/Lipid molar ratio, as extracted from the known DNA concentration and the lipid concentration measured using an enzymatic assay (see Methods). Experimental points are fitted to a Hill function, also used to describe dose-dependent "lethality" [8].

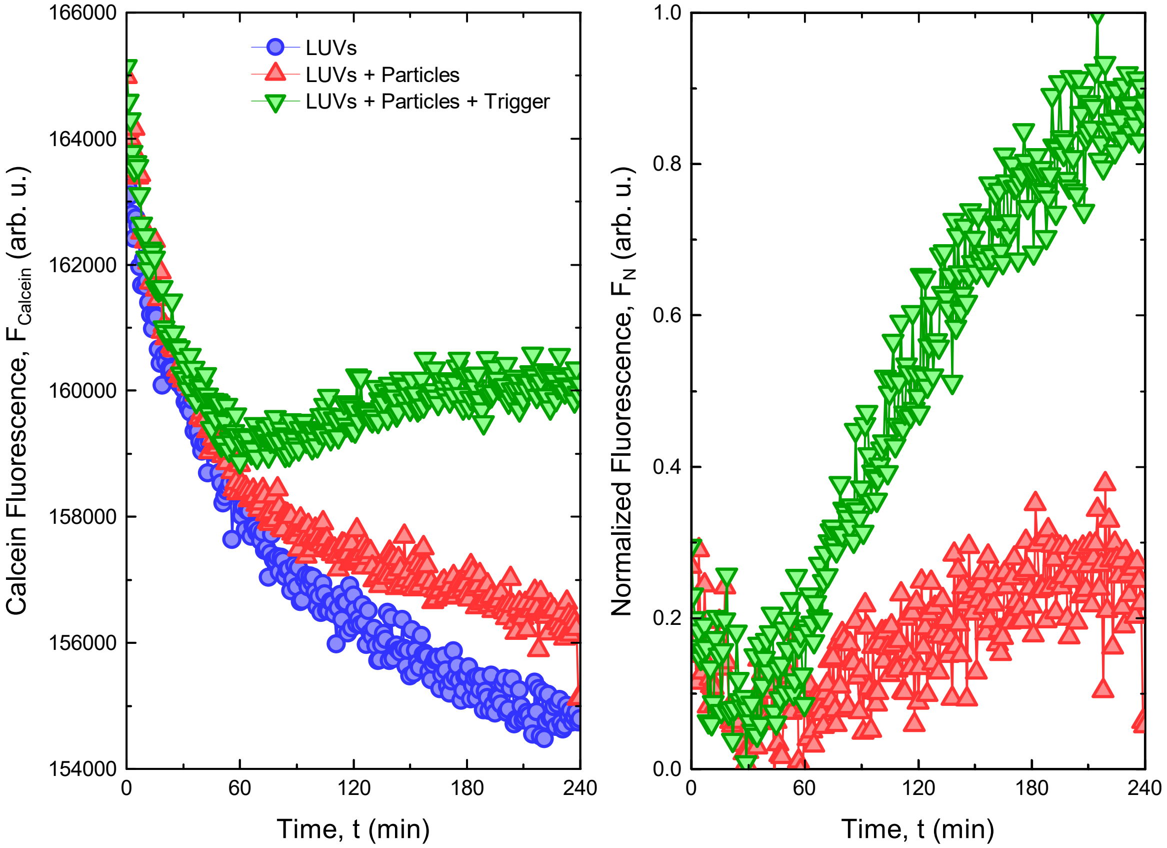

Supplementary Figure 15. **Large Unilamellar Vesicle (LUV) leakage assay with fluorescence spectroscopy.** Triggered particles interact with LUVs and lead to a significant increase in spontaneous release of encapsulated calcein. Inside the vesicles, calcein is highly concentrated and thus its fluorescence signal is self-quenched[9, 10]. Upon release, calcein is diluted, boosting the fluorescence emission. Left: raw fluorescence signal. Right: normalized fluorescence signal after subtracting the trace measured for bare LUVs (blue symbols on the left). The (mass) concentration of LUVs used in the assay was $0.96 \pm 0.12\ \mathrm{g\,L^{-1}}$.

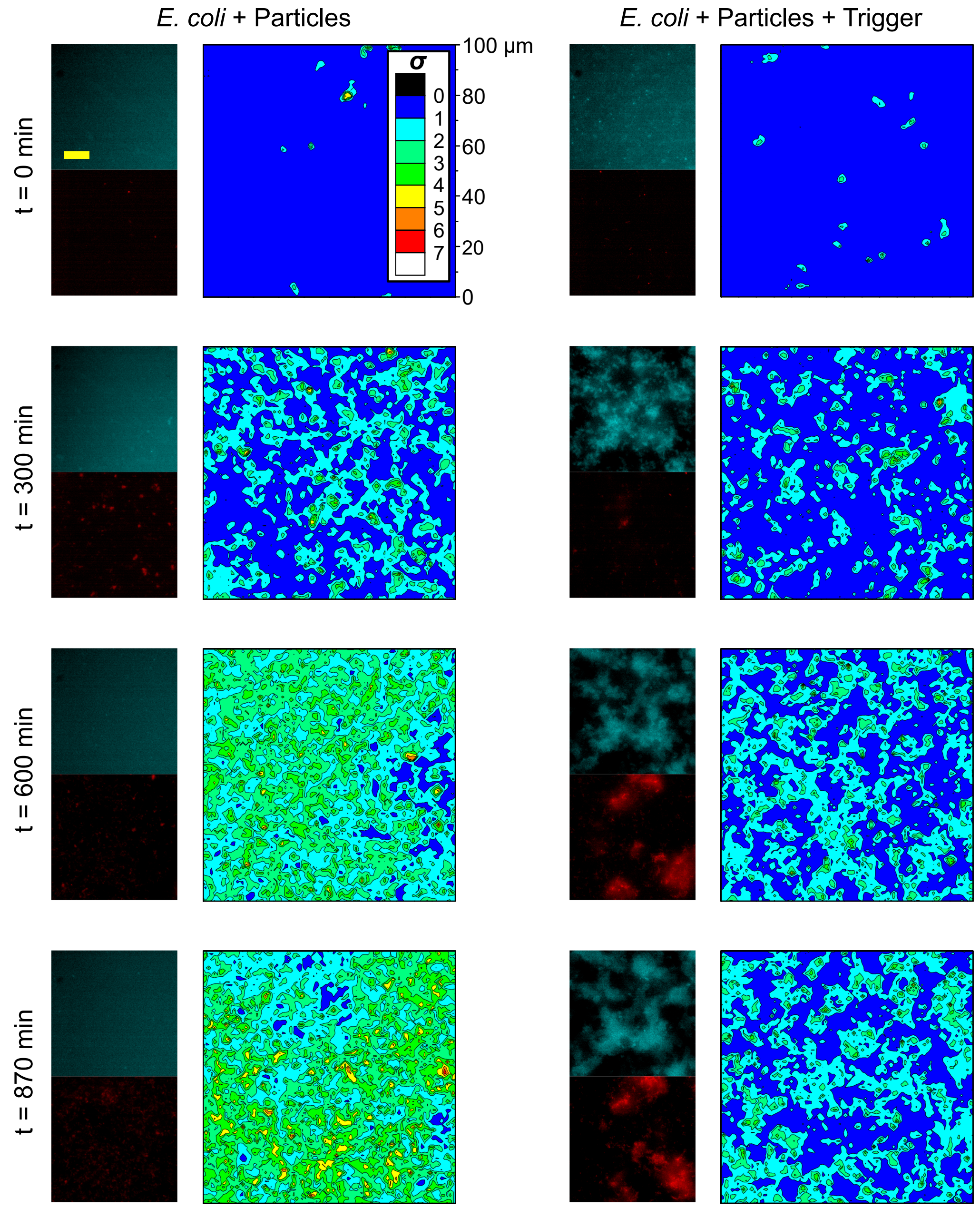

Supplementary Figure 16. **Epifluorescence micrographs and motility colormaps demonstrating the time-dependent entrapment of *E. coli* in a DNA net.** The colormaps indicate values of the motility-parameter σ, defined in the main text and Methods. An increase is observed in both samples with and without the addition of trigger, as a consequence of the steadily increasing number of bacteria, following replication (see Figs 5**e** and 17). Motility is however suppressed in the presence of the trigger, following from the embedding of bacteria in the formed DNA network. Note that, at $t = 0$, σ takes very small values, indicating a negligible contribution from the Brownian motion of particles and the initially present bacteria. The signal progressively emerges from the motion of bacteria as their population grows. The attached micrographs and colormaps represent two independent experiments. In epifluorescence micrographs, particles are shown in cyan (fluorescein), *E. coli* in red (mKate2). Scale bar 20 μm.

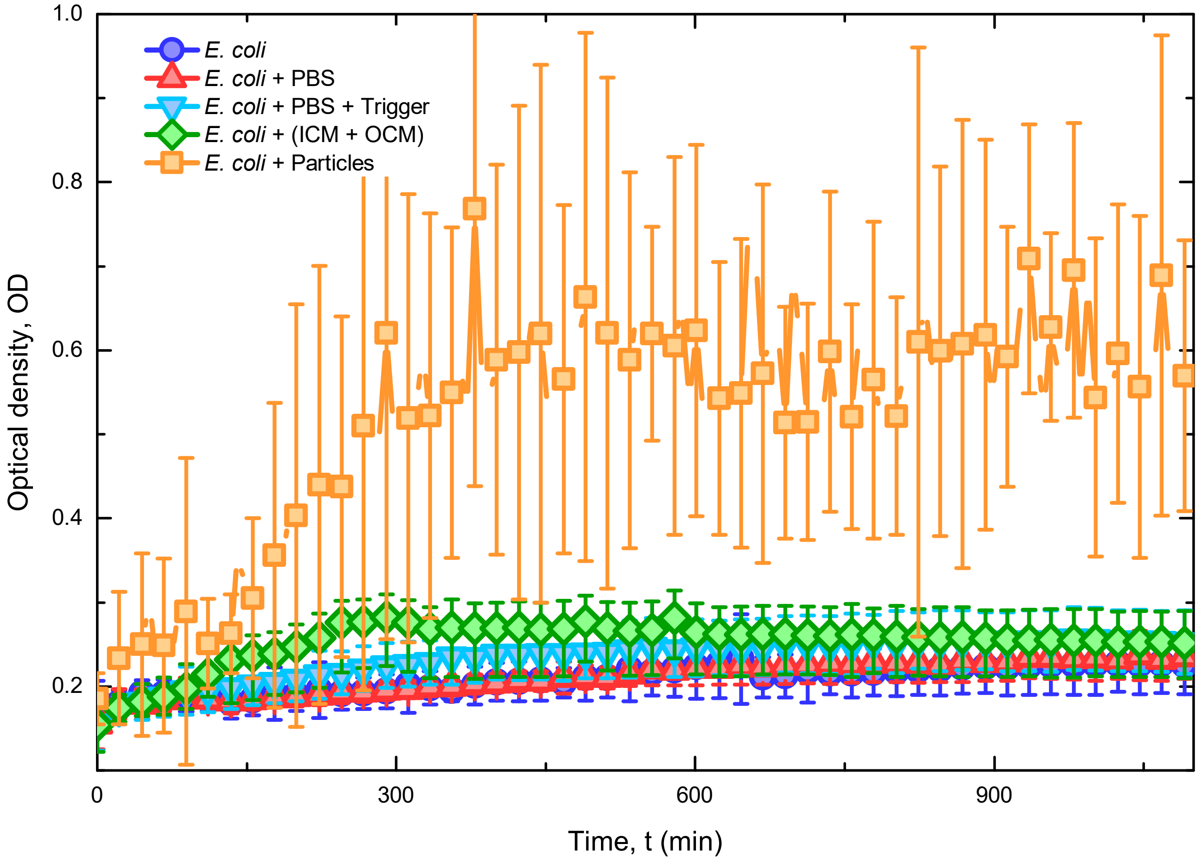

Supplementary Figure 17. **Bacterial growth in various buffers as determined *via* turbidity measurements.** DNA particles trigger a substantial growth and division of *E. coli*, while no growth is observed with non-cholesterolised DNA constructs (Inner Corona Motif = ICM, Outer Corona Motif = OCM, trigger), or DNA-free buffers. These results indicate the ability of *E. coli* to uptake cholesterolised DNA and use it as a source of food. Each experimental point represents the average value of OD ± standard deviation obtained from two independent experiments, each consisting of three independent repeats. Buffer conditions for *E. coli* and DNA constructs are reported in the the relevant Methods section.

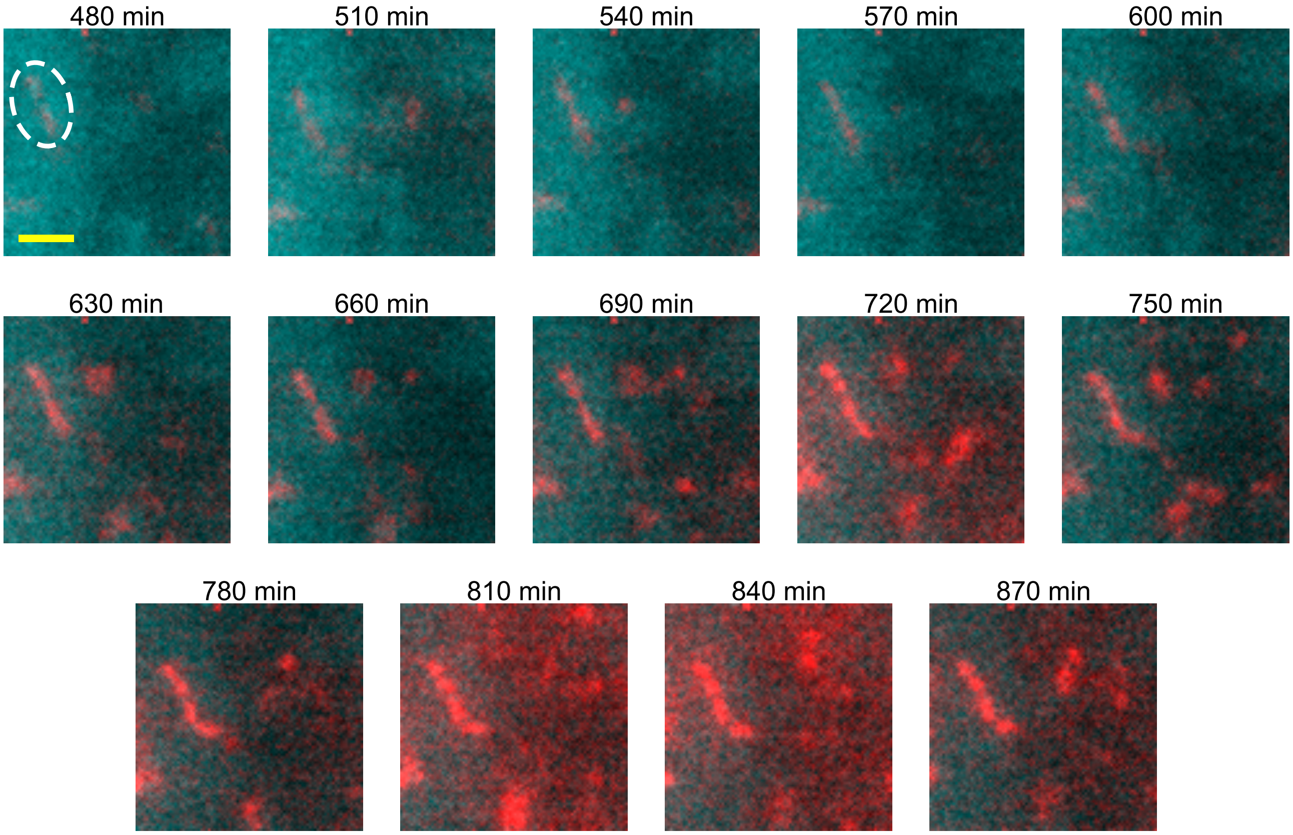

Supplementary Figure 18. ***E. coli* grow while embedded in the amphiphilic DNA network.** Epifluorescence micrographs demonstrating that *E. coli* embedded in the amphiphilic DNA network retain their normal morphology, continue to grow, and do not loose mKate2 fluorescent signal through leakage. This indicates that, while successfully immobilising the cells, the DNA network does not significantly destabilise the bacterial cell wall, which could be a consequence of the protective action of the lipopolysaccharide layer surrounding the outer membrane. The trigger strand was added at time 0. Selected micrographs represent two independent experiments. Core motifs are shown in cyan (fluorescein), *E. coli* in red (mKate2). Scale bar 3 μm.

* l.di-michele@imperial.ac.uk

[1] Rao, C. N. R., Müller, A. & Cheetham, A. K. *Nanomaterials Chemistry: Recent Developments and New Directions* (Wiley-VCH, Weinheim, Germany, 2007), 1 edn.

[2] Zheng, H. *et al.* Observation of single colloidal platinum nanocrystal growth trajectories. *Science* **324**, 1309–1312 (2009).

[3] Viswanatha, R. *et al.* Understanding the quantum size effects in zno nanocrystals. *J. Mater. Chem.* **14**, 661–668 (2004).

[4] Lifshitz, I. M. & Slyozov, V. V. The kinetics of precipitation from supersaturated solid solutions. *J. Phys. Chem. Solids* **19**, 35–50 (1961).

[5] Lifshitz, E. M. & Pitaevskii, L. P. *Landau and Lifshitz Course of Theoretical Physics, Vol. 10* (Butterworth-Heinemann, Oxford, United Kingdom, 1981), 1 edn.

[6] Safari, M. S., Vorontsova, M. A., Poling-Skutvik, R., Vekilov, P. G. & Conrad, J. C. Differential dynamic microscopy of weakly scattering and polydisperse protein-rich clusters. *Phys. Rev. E* **92(4)**, 1–12 (2015).

[7] Mergny, J. L. & Lacroix, L. Analysis of thermal melting curves. *Oligonucleotides* **13(6)**, 515–537 (2003).

[8] Motulsky, H. & Christopoulos, A. *Fitting Models to Biological Data Using Linear and Nonlinear Regression: A Practical Guide to Curve Fitting* (Oxford University Press, Oxford, United Kingdom, 2004), 1 edn.

[9] Allen, T. M. *Liposome Technology, vol. III* (CRC Press, Boca Raton, United States of America, 1984), 1 edn.

[10] Allen, T. M. & Cleland, L. G. Serum-induced leakage of liposome contents. *Biochim. Biophys. Acta* **597**, 418–426 (1980).
