# Supplementary material for: Responsive core-shell DNA particles trigger lipid-membrane disruption and bacteria entrapment
Source: Nat Commun. 2021 Aug 6;12:4743. doi: 10.1038/s41467-021-24989-7 (PMC8346484; doi:10.1038/s41467-021-24989-7)
Supplement: Supplementary file 3 — Reporting Summary [file 41467_2021_24989_MOESM3_ESM.pdf]

nature research

Corresponding author(s): Lorenzo Di Michele

Last updated by author(s): May 29, 2021

Reporting Summary

Nature Research wishes to improve the reproducibility of the work that we publish. This form provides structure for consistency and transparency in reporting. For further information on Nature Research policies, see our Editorial Policies and the Editorial Policy Checklist.

Statistics

For all statistical analyses, confirm that the following items are present in the figure legend, table legend, main text, or Methods section.

n/a	Confirmed	
☐	☒	The exact sample size (n) for each experimental group/condition, given as a discrete number and unit of measurement
☐	☒	A statement on whether measurements were taken from distinct samples or whether the same sample was measured repeatedly
☒	☐	The statistical test(s) used AND whether they are one- or two-sided<br>*Only common tests should be described solely by name; describe more complex techniques in the Methods section.*
☒	☐	A description of all covariates tested
☒	☐	A description of any assumptions or corrections, such as tests of normality and adjustment for multiple comparisons
☐	☒	A full description of the statistical parameters including central tendency (e.g. means) or other basic estimates (e.g. regression coefficient) AND variation (e.g. standard deviation) or associated estimates of uncertainty (e.g. confidence intervals)
☒	☐	For null hypothesis testing, the test statistic (e.g. F, t, r) with confidence intervals, effect sizes, degrees of freedom and P value noted<br>*Give P values as exact values whenever suitable.*
☒	☐	For Bayesian analysis, information on the choice of priors and Markov chain Monte Carlo settings
☒	☐	For hierarchical and complex designs, identification of the appropriate level for tests and full reporting of outcomes
☒	☐	Estimates of effect sizes (e.g. Cohen's d, Pearson's r), indicating how they were calculated

Our web collection on statistics for biologists contains articles on many of the points above.

Software and code

Policy information about availability of computer code

Data collection	NanoDrop 2000/2000c data collection software was used to extract the concentrations of all the reconstituted DNA strands from their absorbance at 260 nm. Agarose gels were imaged using Vision Works 8.20.17096.9551 software. UV melting curves of non-cholesterolized motifs were recorded using Cary WinUV Thermal Application 4.20(468) software. DLS data was collected using Malvern Zetasizer (7.13) software. TEM was performed using Gatan Digital Micrograph 2.32.888.0 software. Confocal images shown in this study were registered using Leica Application Suite Advanced Fluorescence 2.7.3.9723 software. Acquisition of bright field images/videos and epifluorescence images as well as the preparation of protected particles was controlled with an XML (Extensible Markup Language) script generated with a previously written MATLAB (MATLAB R2019b) script. Omega Control 3.00 R3 microplate reader data collection/analysis software was employed to collect the data for the calcein leakage assay from LUVs as well as the study of bacterial growth in a presence of various DNA constructs.
Data analysis	DNA nanostructures were designed using the NUPACK online tool (http://www.nupack.org/). The relative migration distance for samples shown in agarose gel images as well as the melting temperatures of DNA motifs and overhang domains were calculated using custom MATLAB (MATLAB R2019b) scripts. DDM data was analysed with scripts written in MATLAB and Python (Jupyter Notebook 6.0.1). TEM micrographs were processed on Fiji - ImageJ 1.53c including a Bio-Formats 6.5.1 package. Confocal images were analysed using Fiji - ImageJ and MATLAB software. The analysis of E. coli related data (bright field videos and epifluorescence micrographs) was conducted with MATLAB scripts. The rest of the data was processed in OriginPro 9.0. All of the figures were prepared in Inkscape 0.92.4.<br>All the above mentioned custom scripts are available from the corresponding author upon reasonable request.

For manuscripts utilizing custom algorithms or software that are central to the research but not yet described in published literature, software must be made available to editors and reviewers. We strongly encourage code deposition in a community repository (e.g. GitHub). See the Nature Research guidelines for submitting code & software for further information.

Data

Policy information about availability of data

All manuscripts must include a data availability statement. This statement should provide the following information, where applicable:

- Accession codes, unique identifiers, or web links for publicly available datasets
- A list of figures that have associated raw data
- A description of any restrictions on data availability

A fully representative selection of the data underlying these findings can be accessed free of charge at https://doi.org/10.17863/CAM.70338. A detailed description of said data is provided in the repository. Owing to the very large data volume associated to the article, particularly high-resolution microscopy images and high frame-rate videos, it would have been unpractical to upload the complete datasets. These are however available upon request to the corresponding author.

Field-specific reporting

Please select the one below that is the best fit for your research. If you are not sure, read the appropriate sections before making your selection.

☒ Life sciences ☐ Behavioural & social sciences ☐ Ecological, evolutionary & environmental sciences

For a reference copy of the document with all sections, see nature.com/documents/nr-reporting-summary-flat.pdf

Life sciences study design

All studies must disclose on these points even when the disclosure is negative.

Sample size	All the experiments were performed in three replicates unless stated otherwise.<br>No statistical methods were used to predetermine the sample size. Sample sizes were chosen to be three based on experience and previous studies. This ensures that we can notice any errors in the data acquisition process and allows us to quantify the variation in experimentally obtained values for independent samples.
Data exclusions	No data were excluded in the analysis.
Replication	The experimental results shown in the main manuscript, excluding panel d in Figure 4 and panel c in Figure 5 (two independent experiments) are representative of three independent experiments. Acquisition of data underlying the findings shown in the Supplementary Information was performed twice, excluding Figure S17 (two independent experiments, each consisting of three independent repeats).<br>All the attempts at replication were successful and showed fully consistent results.
Randomization	Sample randomization was not required as the methods and nature of this study did not make it prone to human bias.<br>Specifically, with the exception of the data in Figure 4c, all data were analysed with automated scripts that do not take into account sample identity while processing the data. For the case of Figure 4c, the number of vesicles that did not burst as a result of interactions with the DNA particles was counted by hand. This is a straightforward assessment and carries very limited risks associate to human error or bias.
Blinding	Blinding was not required as the identity of the samples was known prior to the measurement.

Reporting for specific materials, systems and methods

We require information from authors about some types of materials, experimental systems and methods used in many studies. Here, indicate whether each material, system or method listed is relevant to your study. If you are not sure if a list item applies to your research, read the appropriate section before selecting a response.

Materials & experimental systems

n/a	Involved in the study	
☒	☐	Antibodies
☒	☐	Eukaryotic cell lines
☒	☐	Palaeontology and archaeology
☒	☐	Animals and other organisms
☒	☐	Human research participants
☒	☐	Clinical data
☒	☐	Dual use research of concern

Methods

n/a	Involved in the study	
☒	☐	ChIP-seq
☒	☐	Flow cytometry
☒	☐	MRI-based neuroimaging
